# Efficacy of depatuxizumab mafodotin (ABT-414) monotherapy in patients with *EGFR*-amplified, recurrent glioblastoma: results from a multi-center, international study

**DOI:** 10.1007/s00280-017-3451-1

**Published:** 2017-10-26

**Authors:** Martin van den Bent, Hui K. Gan, Andrew B. Lassman, Priya Kumthekar, Ryan Merrell, Nicholas Butowski, Zarnie Lwin, Tom Mikkelsen, Louis B. Nabors, Kyriakos P. Papadopoulos, Marta Penas-Prado, John Simes, Helen Wheeler, Tobias Walbert, Andrew M. Scott, Erica Gomez, Ho-Jin Lee, Lisa Roberts-Rapp, Hao Xiong, Earle Bain, Peter J. Ansell, Kyle D. Holen, David Maag, David A. Reardon

**Affiliations:** 1 Brain Tumor Center, Erasmus MC Cancer Institute, Groene Hilledijk 301, 3075 EA Rotterdam, the Netherlands; 2 School of Cancer Medicine, La Trobe University, Melbourne, VIC Australia; 3 Department of Medicine, University of Melbourne, Melbourne, VIC Australia; 4 Department of Neurology and Herbert Irving Comprehensive Cancer Center, Columbia University Medical Center, New York, NY USA; 5 Northwestern University, Chicago, IL USA; 6 NorthShore University Health System, Evanston, IL USA; 7 Department of Neurological Surgery, University of California, San Francisco, San Francisco, CA USA; 8 Department of Medical Oncology, School of Medicine, University of Queensland, Royal Brisbane and Women’s Hospital, Brisbane, Australia; 9 Henry Ford Health System, Detroit, MI USA; 10 University of Alabama at Birmingham, Birmingham, AL USA; 11 South Texas Accelerated Research Therapeutics (START), San Antonio, TX USA; 12 The University of Texas MD Anderson Cancer Center, Houston, TX USA; 13 NHMRC Clinical Trials Centre, University of Sydney, Sydney, NSW Australia; 14 Medical Oncology, Royal North Shore Hospital, Sydney, NSW Australia; 15 AbbVie Inc., North Chicago, IL USA; 16 Dana-Farber Cancer Institute, Boston, MA USA

**Keywords:** ABT-414, Depatuxizumab mafodotin, EGFR, Antibody–drug conjugate, Recurrent glioblastoma

## Abstract

**Purpose:**

Patients with recurrent glioblastoma (rGBM) have a poor prognosis. *Epidermal growth factor receptor* (*EGFR*) gene amplification is present in ~ 50% of glioblastomas (GBMs). Depatuxizumab mafodotin (depatux-m), formerly ABT-414, is an antibody–drug conjugate that preferentially binds cells with *EGFR* amplification, is internalized and releases a potent antimicrotubule agent, monomethyl auristatin F (MMAF). Here we report the safety, pharmacokinetics, and efficacy of depatux-m monotherapy at the recommended Phase 2 dose (RPTD) in patients with *EGFR*-amplified, rGBM.

**Methods:**

M12-356 (NCT01800695) is an open-label study with three escalation and expansion cohorts. Sixty-six patients with *EGFR*-amplified, rGBM were treated with depatux-m monotherapy at 1.25 mg/kg intravenously every 2 weeks. Adults with measurable rGBM, who were bevacizumab-naïve, with *EGFR* amplification were eligible.

**Results:**

Among 66 patients, median age was 58 years (range 35–80). All patients were previously treated with radiotherapy/temozolomide. The most common adverse events (AEs) were eye related (91%), including blurred vision (65%), dry eye (29%), keratitis, and photophobia (27% each). Grade 3/4 AEs occurred in 42% of all patients, and ocular Grade 3/4 AEs occurred in 33% of patients overall. One patient (2%) had a Grade 4 ocular AE. Ocular AEs were manageable and usually resolved once treatment with depatux-m ceased. The objective response rate was 6.8%, the 6-month progression-free survival rate was 28.8%, and the 6-month overall survival rate was 72.5%.

**Conclusion:**

Depatux-m monotherapy displayed frequent but mostly Grade 1/2 ocular toxicities. A PFS6 of 28.8% was observed in this rGBM population, warranting further study.

**Electronic supplementary material:**

The online version of this article (doi:10.1007/s00280-017-3451-1) contains supplementary material, which is available to authorized users.

## Introduction

Glioblastoma (GBM) is the most common malignant brain cancer with an incidence of 2–3 of every 100,000 adults per year. Patients afflicted with GBM have a poor prognosis, with a median survival of 14–16 months from original diagnosis [1, 2]. Many patients will experience recurrent disease (rGBM), and treatment options are limited, with survival under 12 months and rare responses [3]. Six-month progression-free survival rates exceeding 20–25% are considering promising in this setting [4].

Given the dismal survival rates in rGBM, there is an urgent need to develop effective novel therapies. Amplification of the *Epidermal Growth Factor Receptor* (*EGFR*) gene, observed in 50% of GBMs [5–7], creates a tumor-specific target for experimental treatment. About 50% of GBMs with *EGFR* amplification also harbor the *EGFRvIII* deletion variant [8]. Of note, *EGFR* amplification usually remains unchanged at the time of tumor recurrence [9]. Several types of targeted therapies have been used to target EGFR in GBM. Tyrosine kinase inhibitors (TKIs) such as gefitinib and erlotinib have been found to increase PFS in non-small cell lung cancer [10] but have not proven efficacious in GBM, [11–16]. Antibodies that target and bind the extracellular domain of EGFR, such as cetuximab, have shown decreased tumor growth and increased survival in mouse xenograft models [17] but again, did not demonstrate a survival benefit in patients [18]. There are several explanations for the failures of these agents, in particular the absence of the *EGFR* exon 19 deletion and exon 21 mutations that are correlated with activity in NSCLC [19]. Immunotherapy has improved outcomes for many cancers, and the vaccine rindopepimut, which targets *EGFRvIII*, showed promising results in early stage testing in GBM. However, a Phase 3 trial was recently discontinued due to a lack of survival benefit [20]. Numerous ongoing studies are evaluating the potential activity of various immunotherapy agents, including vaccines and immune checkpoint inhibitors, in newly diagnosed and recurrent GBM.

Depatuxizumab mafodotin (depatux-m), formerly ABT-414, is an antibody–drug conjugate (ADC) composed of the EGFR-directed monoclonal antibody, depatuxizumab (depatux), formerly ABT-806, conjugated to the potent antimicrotubule agent monomethyl auristatin F (MMAF, now mafodotin) via a non-cleavable maleimidocaproyl linker [21, 22]. *EGFR* amplification leads to a unique conformation of the EGFR protein that exposes a tumor-specific binding site for depatux-m. This epitope is also exposed in the *EGFRvIII* deletion variant. Once depatux-m enters the cell, MMAF is released, leading to cell death. Depatux-m has limited binding to EGFR in normal tissues and thus does not lead to other toxicities typically associated with other EGFR-targeted therapies, usually dermatological [23]. Preclinical data suggest that depatux-m has potent anti-tumor activity in GBM cell lines and xenograft models [6].

Recently published results from this study show that depatux-m in combination with either chemoradiation or TMZ in both newly diagnosed and recurrent GBM has a tolerable safety and pharmacokinetics (PK) profile [24, 25]. Here, we present efficacy data, including objective response rate (ORR) and PFS6, for depatux-m monotherapy at the recommended Phase 2 dose (RPTD) in patients with recurrent, *EGFR*-amplified GBM.

## Materials and methods

Study M12-356 (NCT01800695) was a multi-center, Phase 1, open-label study designed to evaluate the safety, preliminary efficacy and PK of depatux-m alone or in combination with other treatments in patients with GBM. The trial had three treatment arms: Arm A, depatux-m with radiation therapy (RT) and temozolomide (TMZ) in newly diagnosed GBM; Arm B, depatux-m with TMZ after RT in newly diagnosed or recurrent GBM; and Arm C, depatux-m monotherapy in rGBM. Each arm was composed of a dose escalation and dose expansion cohort [24]. This study was performed in accordance with the 1964 Declaration of Helsinki and its later amendments. All patients provided written informed consent prior to enrollment according to national regulation; the study design was approved by the Institutional Review Board/Ethics Committees of participating institutions.

### Patients

This analysis encompassed 66 patients from Arm C who had *EGFR*-amplified, rGBM and received at least one dose of depatux-m at the RPTD of 1.25 mg/kg. Inclusion and exclusion criteria were as described previously [24]. Only patients with rGBM and centrally confirmed *EGFR* amplification were included. More specifically, patients had Response Assessment in Neuro-Oncology (RANO) defined [26] disease progression which included either: (1) measurable progressive or rGBM as seen by contrast-enhancing MRI and an interval of at least 12 weeks from completion of RT to study entry; (2) progression outside the radiation field; or (3) biopsy or surgically proven disease progression. An MRI with contrast was required within 14 days of Study Day 1, and patients were required to be on a stable or decreasing dose of corticosteroids for at least 5 days prior to the scan. Patients were ineligible if: they had received bevacizumab as prior treatment for rGBM, had a secondary GBM, or had been exposed to prior EGFR therapy for GBM, including EGFRvIII-specific immunotherapies.

### Study design

Study design of M12-356 has been described previously [24, 25]. The primary objective was to determine the ORR [partial response (PR) + complete response (CR)]. The secondary objectives were to determine the PFS6, PFS, OS, and safety and tolerability of depatux-m.

### Treatment regimen

The RPTD of depatux-m monotherapy was determined previously as 1.25 mg/kg via intravenous (IV) infusion every 2 weeks [25]. All patients received 1.25 mg/kg of depatux-m via intravenous infusion over 30–40 min on Days 1 and 15 of a 28-day cycle (Supplementary Fig. 1). Radiographic assessment of disease progression was performed before every other cycle. Treatment was intended to continue until either intolerable toxicity or disease progression as assessed locally by the investigator using RANO criteria [26]. Central review was not performed. Depatux-m dosing could be reduced to 1.0 or 0.5 mg/kg for Grade 3/4 toxicities. Re-escalation was permitted.

### Pharmacokinetics

Serum samples for the determination of depatux-m concentrations were collected before and immediately after depatux-m infusions on Day 1 of Cycles 1 and 2, and before depatux-m infusions on Day 15 of Cycles 1 and 2. Serum samples for the determination for anti-drug antibody (ADA) were collected biweekly before each depatux-m infusion up to Day 1 of Cycle 3 and once every four weeks before depatux-m infusion in the subsequent cycles. For patients who were able to return to the clinic for the follow-up visit, ADA samples were also collected approximately 35 days after the last depatux-m infusion.

Depatux-m serum concentrations and ADA titers were determined using validated electrochemiluminscence immunoassays [24]. The depatux-m concentrations in the Arm C expanded cohort were compared to those in the Arm C dose escalation cohort only with intensive pharmacokinetic sampling [24].

### Tumor molecular characterization

Molecular characterization of archival tumor tissue, including testing performed to determine *EGFR* expression, amplification, and *EGFRvIII* mutation status before protocol therapy was performed as described previously [24]. Briefly, fluorescence *in situ* hybridization (FISH) was used to detect locus-specific *EGFR* amplification. Two probes were employed: Vysis Locus Specific Identifier (LSI) EGFR SpectrumOrange Probe, and Vysis Chromosome Enumeration Probe (CEP) 7 SpectrumGreen Problem (Abbott Laboratories, Abbott Park, IL, USA). To call a tumor *EGFR* amplified, the sample should show ≥ 15% tumor cells with an EGFR/CEP 7 ratio ≥ 2.

### Statistical analysis

Descriptive statistics were provided for patient demographic variables. Safety/toxicity summaries were provided for all patients who received at least one dose of depatux-m. Frequencies of adverse events (AEs) were tabulated by the National Cancer Institute Common Terminology Criteria for Adverse Events (NCI CTCAE, version 4.1) and listed by MedDRA (version 19) system organ class and preferred term. Responses were assessed per RANO criteria. The primary efficacy endpoint was objective response rate (ORR, complete response (CR) and partial response (PR)) and was determined for patients with measurable disease at baseline. The secondary endpoints included PFS6, PFS, OS, safety and tolerability. PFS was defined as the time period from the first dose of depatux-m to RANO-defined disease progression or date of death, if disease progression did not occur. OS was determined from the time of first dose of depatux-m to death from any cause. Ninety-five percent confidence interval (CI) was constructed for the estimated ORR (determined from the exact binomial distribution), PFS, and OS. The Greenwood formula was used to calculate the confidence limits for the quartiles of survival distribution (PFS and OS).

## Results

### Patient characteristics

As of 15 March 2017, enrollment was completed with 66 patients. The median age was 58 years. Forty-one percent were women and 59% were men. All patients had *EGFR*-amplified rGBM, and were previously treated with RT/TMZ (Table 1). Thirty-one patients (47%) received depatux-m as the first treatment after initial RT/TMZ. Thirty-one (47%) had tumors which harbored an *EGFRvIII* mutation (Table 1), which is similar to previously reported mutation rates of 50% in patients with *EGFR*-amplified GBM [8].

**Table 1:** Patient demographics

Characteristics	*N* = 66 *n* (%)
Gender	
Female	27 (41)
Male	39 (59)
Median age, years (range)	58 (35–80)
Karnofsky performance status, baseline	
100	11 (17)
90	26 (39)
80	20 (30)
70	9 (14)
Prior surgeries	
0	2 (3)
1	30 (46)
2	29 (44)
3	5 (8)
Prior therapies	
Radiation therapy	66 (100)
TMZ	66 (100)
Experimental therapy	11 (17)
Lomustine	6 (9)
Procarbazine	4 (6)
Carboplatin	3 (5)
Gliadel wafers	2 (3)
Carmustine	1 (2)
MGMT methylation status	
Methylated	5 (8)
Unmethylated	16 (24)
Unknown	45 (68)
*EGFRvIII* mutation status	
Positive	31 (47)
Negative	34 (51)
Unknown^a^	1 (2)

^a^Not enough tissue available for testing

### Safety of depatux-m

All patients received depatux-m at the RPTD of 1.25 mg/kg [25]. Sixty-four of 66 patients experienced at least one AE (Table 2). Nearly, all patients (91%) experienced at least one ocular AE. The most frequent included blurred vision (65%) and dry eye (29%). The most common non-ocular event was fatigue (33%).

**Table 2:** All adverse events (AEs)

Adverse events	*N* = 66 *n* (%)
All AEs (≥ 25% of patients)	64 (97)
Non-ocular	
Fatigue	22 (33)
Headache	19 (29)
Ocular	60 (91)
Vision blurred	43 (65)
Dry eye	19 (29)
Keratitis	18 (27)
Photophobia	18 (27)
Eye pain	17 (26)

Forty-two percent of patients experienced a Grade 3/4 AE, with ocular Grade 3/4 adverse events (AEs) due to microcystic keratopathy being the most common (35%, Table 3). Ocular-related Grade 3/4 AEs included keratitis (17%), corneal epithelial microcysts (8%), blurred vision (5%), and reduced visual acuity (5%). Non-ocular Grade 3/4 AEs occurred in 15% of patients. A further breakdown of all ocular AEs by grade (1/2 vs. 3/4, Supplementary Table 1) showed that the majority were Grade 1/2. Only 1 Grade 4 AE of reduced visual acuity was observed. A serious AE was observed in 36% of patients (Supplementary Table 2), with seizure (9%) occurring most frequently. Two serious AEs were assessed by the investigator as having a reasonable possibility as being attributable to depatux-m. These included one case of seizure and one case of cerebrovascular accident, which are not uncommon in patients with rGBM.

**Table 3:** Grade 3/4 AEs having a reasonable possibility as being depatux-m-related

Grade 3/4 AEs	*N* = 66 *n* (%)
All Grade 3/4 AEs	28 (42)
Ocular	23 (35)
Keratitis	11 (17)
Corneal epithelial microcysts	5 (8)
Vision blurred	3 (5)
Visual acuity reduced	3 (5)
Dry eye	2 (3)
Ulcerative keratitis	2 (3)
Lacrimation increased	1 (2)
Intraocular pressure increased	1 (2)
Non-ocular	9 (15)
Alanine aminotransferase increased	1 (2)
Cerebrovascular accident	1 (2)
Fatigue	1 (2)
Left ventricular dysfunction	1 (2)
Lymphocyte count decreased	1 (2)
Muscular weakness	1 (2)
Neutrophil count decreased	1 (2)
Proteinuria	1 (2)
Seizure	1 (2)

Per investigator assessment

All patients had discontinued depatux-m at the time of analysis, the majority for disease progression (85%). Two patients discontinued for an AE related to progression, and eight patients (12%) discontinued for an AE unrelated to disease progression. These included four patients with ocular side effects, two with thrombocytopenia, one with proteinuria, and one with left-sided muscle weakness. Interruption of depatux-m dosing occurred in 33/66 patients (50%), with the most common reason for interruption due to ocular side effects in 25/66 patients (38%). Finally, 21 patients (32%) underwent a dose reduction of depatux-m due to an AE, with ocular AEs again the most common reason for reduction in 19/66 patients (29%). Fifty-six patients (85%) died during the course of the study.

### Resolution of ocular side effects

**Fig. 1:**
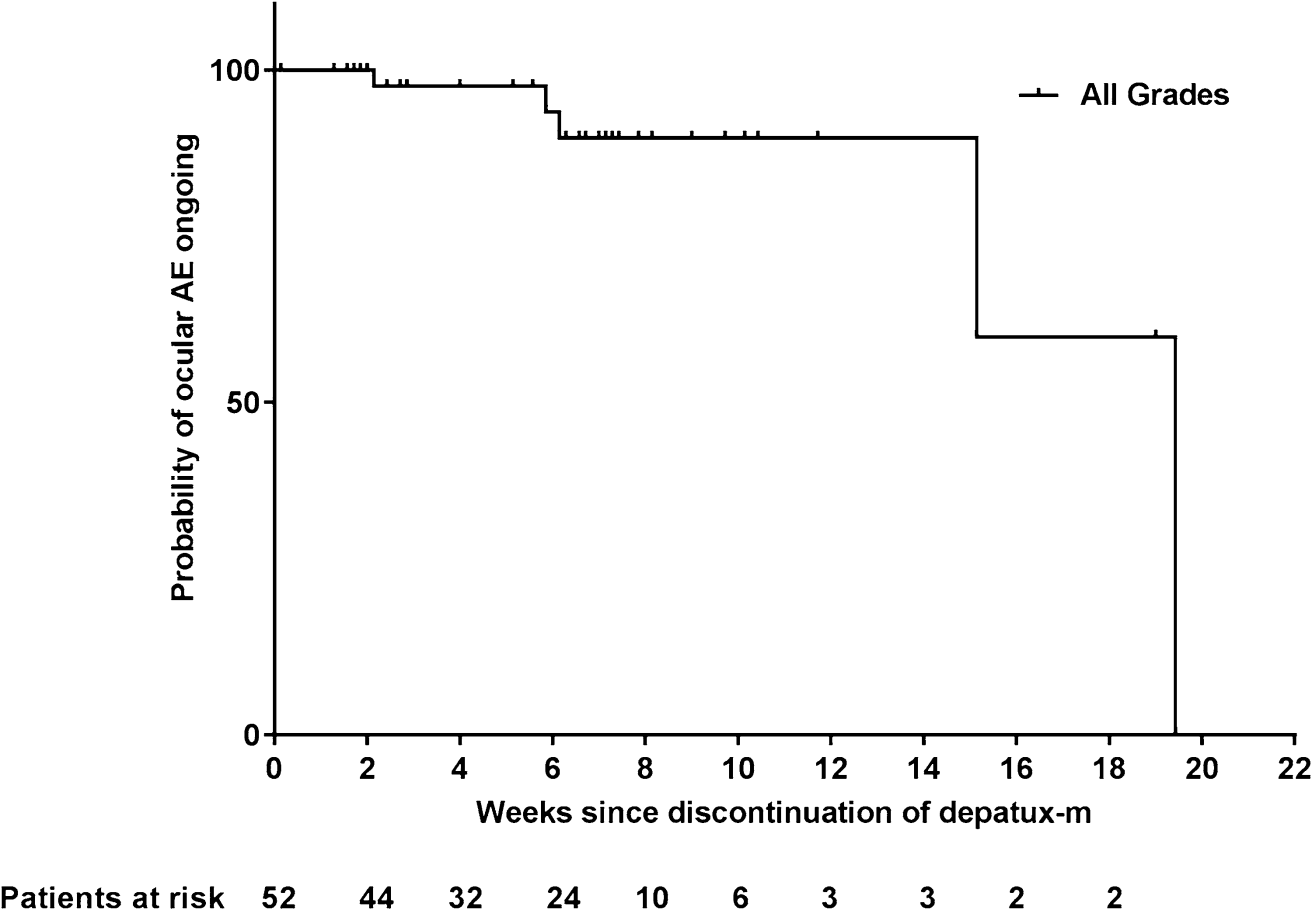
Kaplan–Meier curve of time to resolution of ongoing ocular AEs (all grades) in patients after discontinuation of depatux-m. Time to resolution was defined as the number of days from the last dose date to the last end date of all ocular AEs

As mentioned, ocular side effects were very common in patients. The type and severity of ocular AEs varied, but all were attributed to generalized microcystic keratopathy, which is observed with some types of ADCs (see Discussion). Although the ocular side effects were common, they resulted in treatment discontinuation in only 4 patients (6%). The median time to onset of any ocular side effect was 3.2 weeks (95% CI 2.6, 3.6), as determined from all 66 patients. There was a trend toward reversibility of ocular side effects (Fig. 1); however, a precise definition of a median time to resolution could not be established, due to confounding factors including time on study.

### Pharmacokinetics

The PK of depatux-m in the Arm C expanded cohort was consistent with that in the Arm C dose escalation cohort [25] for both Cycle 1 and Cycle 2 (Supplementary Fig. 2). No ADA was detected or confirmed in any sample during therapy (*n* = 60 patients with at least one post-treatment ADA result) or at final follow-up visit (*n* = 13 patients with ADA results at final follow-up, which was not mandated).

### Efficacy of depatux-m

**Fig. 2:**
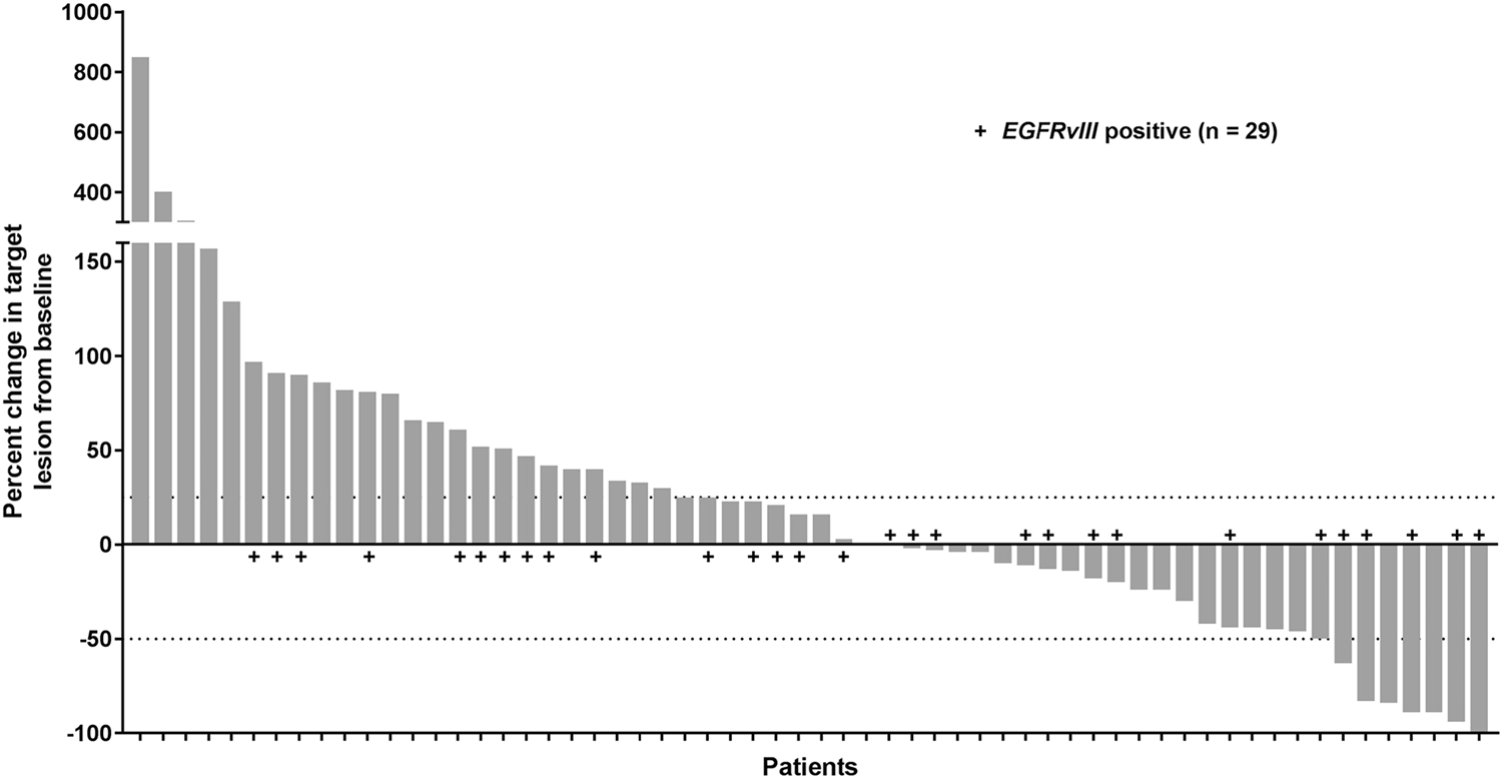
The percent change in target lesion from baseline are shown for 60/66 patients who had at least one post-baseline measurement. Best tumor percent change is defined as the maximum reduction/minimum increase from baseline in tumor size. Values were determined per investigator measurements

**Fig. 3:**
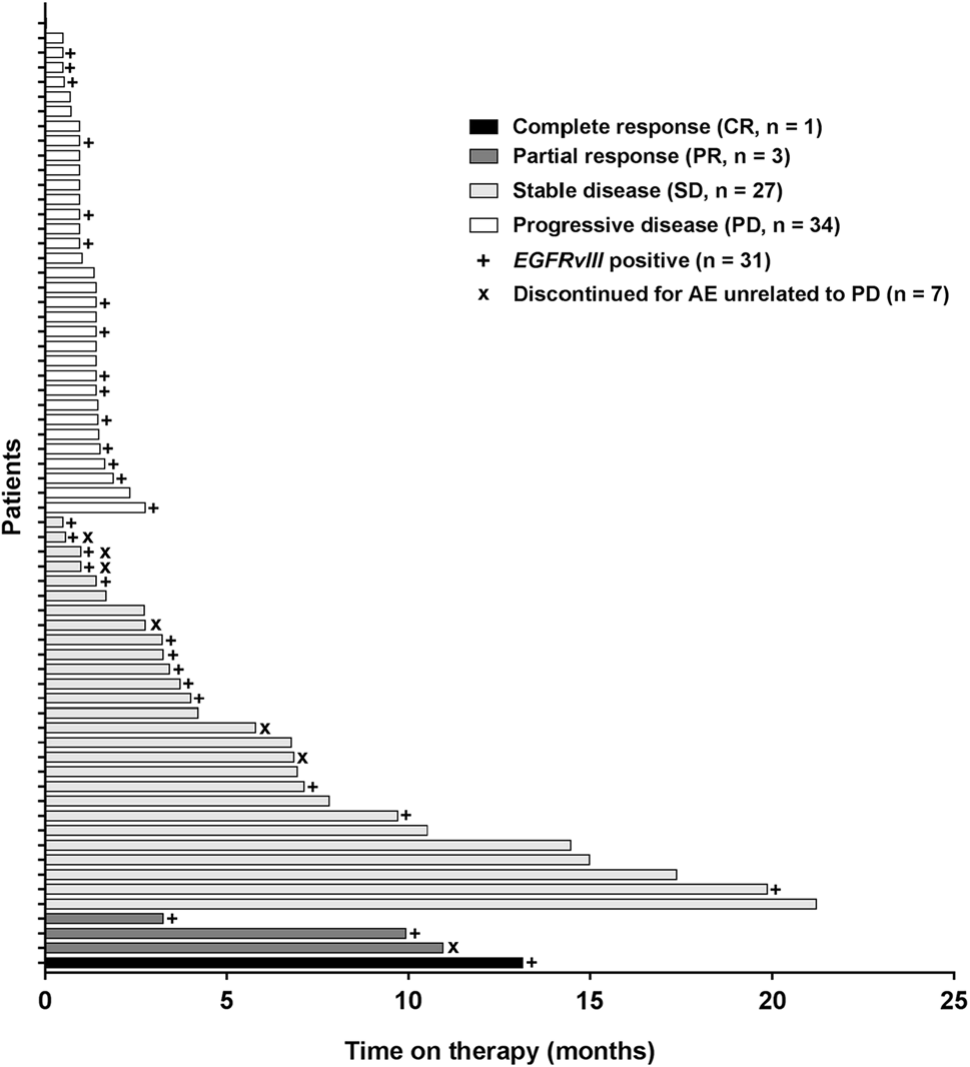
The best responses as determined by the investigator using RANO criteria and time on depatux-m therapy are shown for 65/66 patients with available data. One patient had a baseline assessment but discontinued before the first follow-up, and is not included in this analysis

Sixty of 66 patients had at least one post-baseline assessment allowing determination of change in tumor size (Fig. 2). Per RANO criteria, a best response of stable disease (SD) was observed in 27/66 patients (41%) and 34/66 patients (52%) had a best response of progressive disease (PD, Fig. 3). Of patients with measurable disease at baseline, the ORR was 6.8% (1/59 CR, 3/59 PR, 95% CI 1.9%, 16.5%). The median duration of response in 66 patients was 6.7 months (95% CI 1.6, 8.1).

The PFS6 was 28.8% (95% CI 18.5, 39.9%) and median PFS was 1.7 months (95% CI 1.4, 3.3). The OS6 was 72.5% (95% CI 60.0, 81.7%) and median OS was 9.3 months (95% CI 6.6, 11.7). For patients with *EGFRvIII* mutation (*n* = 29 patients with available response data), the PFS6 was 17.2% (95% CI 6.3%, 32.7%) and median PFS was 1.6 months (95% CI 1.4, 3.3).

## Discussion

Depatux-m monotherapy administered at the RPTD of 1.25 mg/kg in patients with *EGFR*-amplified rGBM demonstrated a PFS6 of 28.8% and OS6 of 72.5%, benchmarks [27] that suggest this drug could show benefits above current standard of care agents. These results, combined with a similar PFS6 of 27.1% in patients with *EGFR*-amplified rGBM treated with depatux-m alone or with TMZ (*n* = 126, which includes patients from this analysis and patients from Arm B) [28], suggest that further investigation of depatux-m in this population is warranted. Ten patients remained on treatment for more than 9 months (Fig. 3), suggesting that despite the ocular side effects, treatment was tolerated for a prolonged period of time. All 10 patients experienced typical ocular AEs, which were mainly Grade 1/2. Six of the ten had Grade 3 keratitis, one had Grade 3 corneal microcysts, and one had Grade 3 reduced visual acuity. One patient had proteinuria and one patient had significant neutropenia, both of which were managed by depatux-m dose interruption and the latter also by dose reduction.

The occurrence of microcystic keratopathy is a very predictable side effect in treatment with ADCs, particularly those with the MMAF toxin [29, 30]. It is not clear why certain ADC payloads induce such specific eye sensitivity, but it could be related to drug accumulation within various ocular tissues. Ocular side effects have been observed in the other arms of this study as well [24, 25]. Ocular side effects were generally manageable with dexamethasone eye drops, corneal bandages, dose reductions, and delays. Other prophylactic measures are being investigated, but have not yet been fully evaluated. Although ocular side effects were frequent, the majority of patients (56%, Supplementary Table 1) experienced only Grade 1/2 side effects, and only 6% of patients discontinued due to an ocular AE. Based on the details of the ten patients who had SD for more than 9 months mentioned above, the severity of ocular side effects did not correlate with time on therapy. Side effects did improve upon treatment discontinuation; however, the median time to resolution was unreliable based on data for a limited number of patients and is thus confounded by competing risks. These include patients who discontinued follow-up for progressive disease, initiated alternative therapies, or died, all of which led to a high censoring rate. Of note, six patients experienced complete resolution of ocular side effects before completion of study treatment.

Additionally, in this *EGFR*-amplified population, *EGFRvIII* mutation did not seem to further differentiate responders from non-responders, or patients likely to experience a PFS event within 6 months. Increased patient numbers are required to further understand the impact that depatux-m may have on the *EGFRvIII* vs. *EGFR* wild-type-amplified populations.

To conclude, we observed in this multicenter, dose expansion study that depatux-m monotherapy administered at the RPTD in patients with *EGFR*-amplified, rGBM demonstrated promising efficacy and manageable toxicity, indicating that further study of this novel targeted therapy in GBM is justified. Two other global, randomized trials are ongoing: depatux-m or placebo + RT/TMZ in *EGFR*-amplified, newly diagnosed GBM (INTELLANCE 1, NCT02573324); and depatux-m vs. depatux-m + TMZ vs. TMZ/lomustine in *EGFR*-amplified, rGBM has completed accrual with results expected in late 2017 (EORTC 1410-BTG, INTELLANCE 2, M14-483, NCT02343406).

## Electronic supplementary material

Below is the link to the electronic supplementary material.

Supplementary material 1 (PPTX 62 KB)

Supplementary material 2 (DOCX 17 KB)
