# Supplementary material for: Efficacy of depatuxizumab mafodotin (ABT-414) monotherapy in patients with EGFR-amplified, recurrent glioblastoma: results from a multi-center, international study
Source: Cancer Chemother Pharmacol. 2017 Oct 26;80(6):1209–17. doi: 10.1007/s00280-017-3451-1 (PMC5686264; doi:10.1007/s00280-017-3451-1)
Supplement: Supplementary file 1 — Supplementary material 1 (PPTX 62 KB) [file 280_2017_3451_MOESM1_ESM.pptx]

## Slide 1
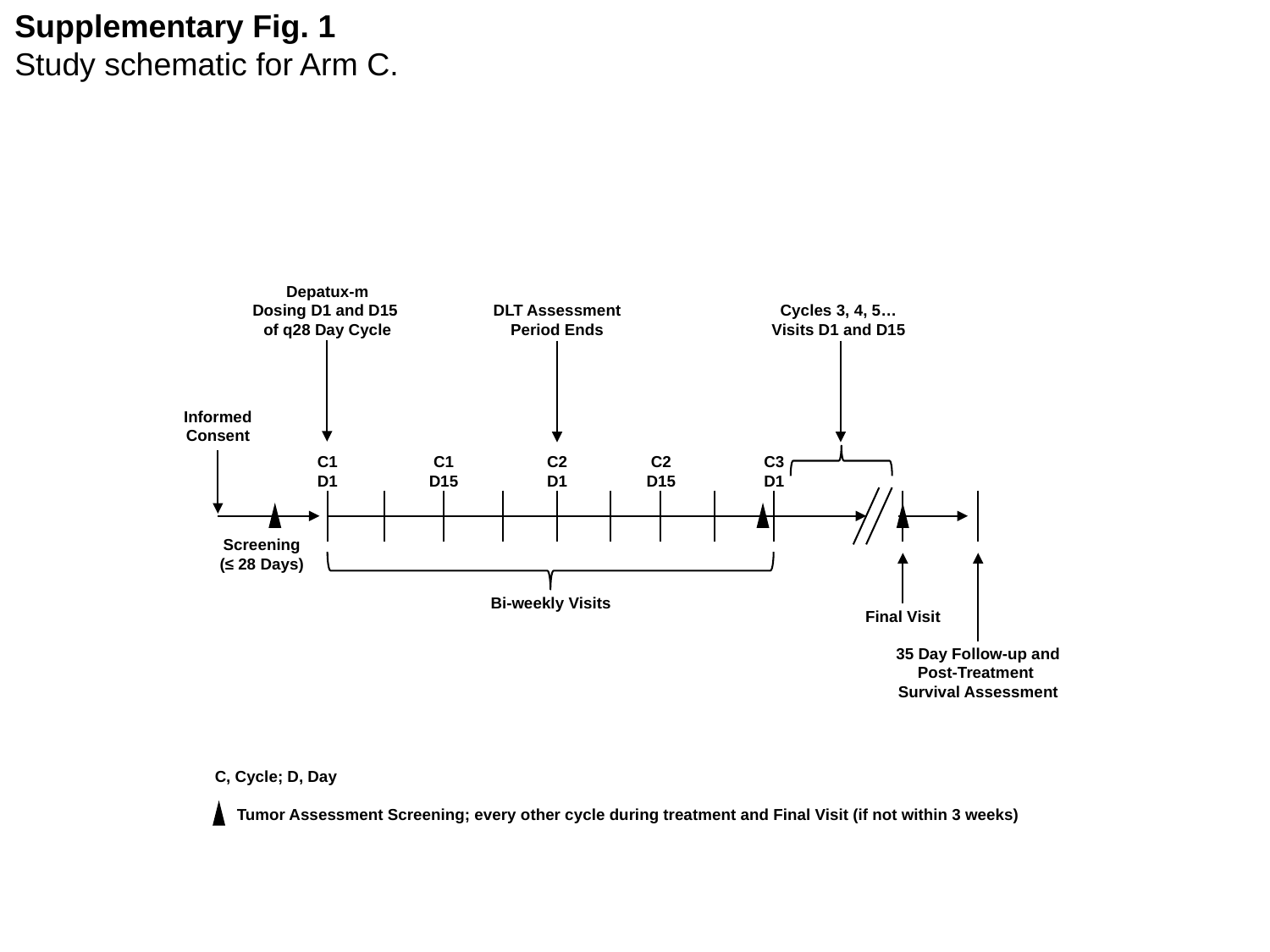

Supplementary Fig. 1
Study schematic for Arm C.
Depatux-m
Dosing D1 and D15
of q28 Day Cycle
DLT Assessment Period Ends
Cycles 3, 4, 5…
Visits D1 and D15
Informed Consent
C1
D1
C1
D15
C2
D1
C2
D15
C3
D1
Screening
(≤ 28 Days)
Bi-weekly Visits
Final Visit
35 Day Follow-up and Post-Treatment
Survival Assessment
C, Cycle; D, Day
 Tumor Assessment Screening; every other cycle during treatment and Final Visit (if not within 3 weeks)

## Slide 2
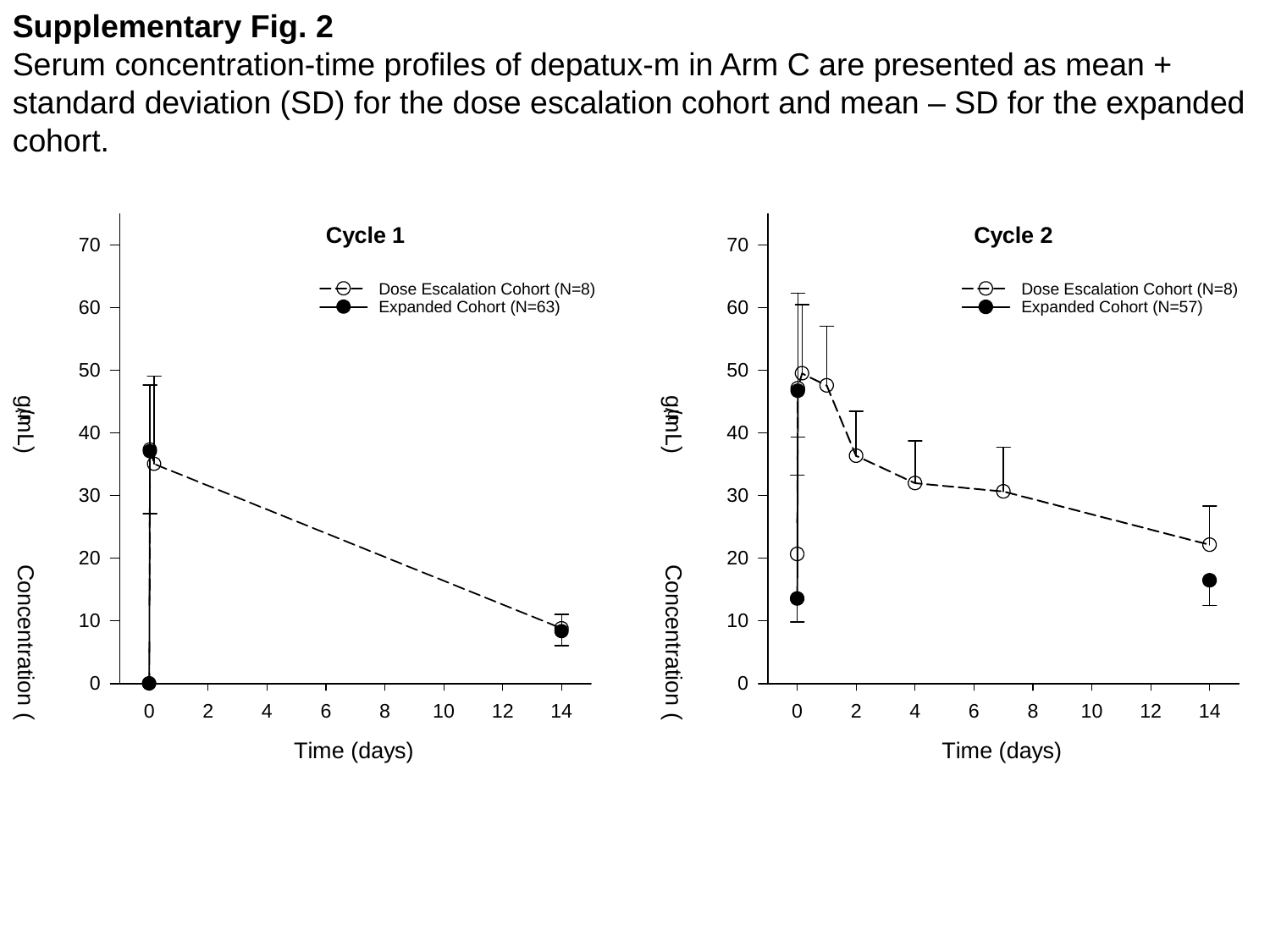

Supplementary Fig. 2
Serum concentration-time profiles of depatux-m in Arm C are presented as mean + standard deviation (SD) for the dose escalation cohort and mean – SD for the expanded cohort.
