# Supplementary material for: Efficacy of depatuxizumab mafodotin (ABT-414) monotherapy in patients with EGFR-amplified, recurrent glioblastoma: results from a multi-center, international study
Source: Cancer Chemother Pharmacol. 2017 Oct 26;80(6):1209–17. doi: 10.1007/s00280-017-3451-1 (PMC5686264; doi:10.1007/s00280-017-3451-1)
Supplement: Supplementary file 2 — Supplementary material 2 (DOCX 17 KB) [file 280_2017_3451_MOESM2_ESM.docx]

Supplementary Table 1. Breakdown of ocular AEs by grade

| **Ocular AEs** | **N = 66**  **n (%)** |
| --- | --- |
| **All ocular AEs**  **Gr 1/2**  **Gr 3/4** | **37 (56)**  **23 (35)** |
| Vision blurred  Gr 1/2  Gr 3 | 40 (61)  3 (5) |
| Eye pain  Gr 1/2 | 17 (26) |
| Dry eye  Gr 1/2  Gr 3 | 17 (26)  2 (3) |
| Photophobia  Gr 1/2 | 18 (27) |
| Keratitis  Gr 1/2  Gr 3 | 7 (11)  11 (17) |
| Foreign body sensation  Gr 1/2 | 12 (18) |
| Lacrimation increased  Gr 1/2  Gr 3 | 8 (12)  1 (2) |
| Corneal epithelial microcysts  Gr 1/2  Gr 3 | 5 (8)  5 (8) |
| Eye irritation  Gr 1/2 | 8 (12) |
| Intraocular pressure increased  Gr 1/2  Gr 3 | 4 (6)  1 (2) |
| Visual acuity reduced  Gr 1/2  Gr 3  Gr 4 | 1 (2)  2 (3)  1 (2) |
| Ulcerative keratitis  Gr 1/2  Gr 3 | 1 (2)  2 (3) |

Ocular AEs shown here occurred in > 10% patients overall and/or > 1 patient with a Grade 3 or 4 AE

Supplementary Table 2. Serious AEs

| **Serious AEs (> 1 patient)** | **N = 66**  **n (%)** |
| --- | --- |
| **All Serious AEs** | **24 (36)** |
| Seizure | 6 (9) |
| Hydrocephalus | 2 (3) |
| Partial seizures | 2 (3) |
| Syncope | 2 (3) |
